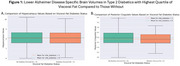# Brain Structure in Type 2 Diabetics with High Visceral Adiposity

**DOI:** 10.1002/alz70856_106639

**Published:** 2026-01-09

**Authors:** Cyrus A. Raji, Somayeh Meysami, Soojin Lee, Saurabh Garg, Nasrin Akbari, Rodrigo Solis Pompa, Ahmed Gouda, Thanh Duc Nguyen, Saqib Basar, Yosef Gavriel Chodakiewitz, David A. Merrill, Amar Patel, Daniel J. Durand, Sam Hashemi

**Affiliations:** ^1^ Mallinckrodt Institute of Radiology, Washington University in St. Louis, St. Louis, MO, USA; ^2^ Pacific Brain Health Center, Pacific Neuroscience Institute and Foundation, Santa Monica, CA, USA; ^3^ Saint John's Cancer Institute at Providence Saint John's Health Center, Santa Monica, CA, USA; ^4^ Vigilance Health Imaging Network Inc., Vancouver, BC, Canada; ^5^ Prenuvo, Palo Alto, CA, USA

## Abstract

**Background:**

Dementia, including Alzheimer's Disease (AD), is preventable, with modifiable risk factors accounting for up to 45% of cases. Previous research has shown that higher visceral fat is related to atrophy in brain regions vulnerable to early AD pathology, such as the hippocampus and posterior cingulate gyrus. This study investigated the confluence of high visceral fat with type 2 diabetes mellitus on brain volume loss, a critical biomarker of neurodegeneration.

**Method:**

A cross‐sectional study of 4,213 adults (mean age 55.39 ± 12.84 years, 57.3% women) underwent whole‐body MRI at 1.5T across sites in Vancouver, Canada; San Francisco Bay Area, California; and Minneapolis, Minnesota. Scanner models included Siemens Espree and Aera. The protocol included whole‐body sagittal T1‐ and T2‐weighted images, coronal STIR, and axial T1 images with Dixon technique, enabling visual identification and quantification of visceral adipose tissue (VAT) and subcutaneous adipose tissue (SAT). Deep learning models measured visceral fat and brain volumes computed from regional segmentations using Fastsurfer. Type 2 diabetes mellitus history was obtained through participant self‐report. Brain volumes in brain parenchyma, hippocampus, posterior cingulate, and precuneus were compared in diabetics with the highest quartile of VAT (*n* = 144) to those without, using ANCOVA adjusted for age, sex, and total intracranial volume.

**Result:**

Persons with type 2 diabetes mellitus were significantly older (mean age 61.5 ± 11.3 years; *p* <.001), more likely to be obese (mean BMI 33.3 ± 5.8; *p* <.001), and predominantly male (71%; *p* <.001). Type 2 diabetics with the highest quartile of VAT showed significantly lower brain parenchymal volumes (F=632, *p* <.0001), reduced hippocampal volume (Figure 1A; F=376.2, *p* = .012), and decreased posterior cingulate gyrus volume (Figure 1B; F=336.9, *p* = .000013). No significant difference was observed in precuneus volume (*p* = .54).

**Conclusion:**

Individuals with type 2 diabetes mellitus and higher amounts of visceral fat exhibited lower volumes in whole brain parenchyma and regions vulnerable to Alzheimer's disease. These findings suggest that type 2 diabetes and visceral obesity may be important targets for secondary prevention of AD as early as midlife. The public health implications are significant, as these conditions are modifiable through behavioral and pharmacological interventions, potentially offering a promising approach to mitigating AD risk.